# Renal function is independently associated with circulating betatrophin

**DOI:** 10.1371/journal.pone.0173197

**Published:** 2017-03-03

**Authors:** Lukas Maurer, Franziska Schwarz, Antje Fischer-Rosinsky, Nina Schlueter, Sebastian Brachs, Matthias Möhlig, Andreas Pfeiffer, Knut Mai, Joachim Spranger, Thomas Bobbert

**Affiliations:** 1 Dept. of Endocrinology, Diabetes and Nutrition, Charité-Universitätsmedizin Berlin, Berlin, Germany; 2 Experimental and Clinical Research Center, Charité-Universitätsmedizin Berlin and Max-Delbrück Zentrum, Berlin-Buch, Germany; 3 Charité Center for Cardiovascular Research (CCR), Charité-Universitätsmedizin Berlin, Berlin, Germany; 4 DZHK (German Centre for Cardiovascular Research), Partner Site Berlin, Berlin, Germany; 5 Dept. of Clinical Nutrition, German Institute of Human Nutrition, Nuthetal, Germany; Medizinische Universitat Innsbruck, AUSTRIA

## Abstract

**Objective:**

Betatrophin has been identified as a marker linking liver with beta cell function and lipid metabolism in murine models. Until now, the regulation of circulating betatrophin in humans is not entirely clear. We here analyzed the relation of betatrophin levels to phenotypes of the metabolic syndrome and speculated that renal function might influence circulating betatrophin levels and explain age-dependent changes of betatrophin.

**Subjects:**

We analyzed blood samples from 535 individuals participating in the Metabolic Syndrome Berlin Potsdam study.

**Results:**

In a crude analysis we found a positive correlation between betatrophin levels and HbA1c (r = 0.24; p < 0.001), fasting glucose (r = 0.20; p < 0.001) and triglycerides (r = 0.12; p = 0.007). Furthermore betatrophin was positively correlated with age (r = 0.47; p <0.001), systolic blood pressure (r = 0.17; p < 0.001), intima media thickness (r = 0.26; p < 0.001) and negatively correlated with CKD-EPI eGFR (r = -0.33; p < 0.001) as an estimate of renal function. Notably, eGFR remained highly associated with betatrophin after adjustment for age, waist circumference, gender, HbA1c and lipid parameters in a multivariate linear regression model (β = -0.197, p< 0.001).

**Conclusions:**

Our data suggest that circulating levels of betatrophin depend on age, gender, waist circumference, total/HDL cholesterol ratio and renal function. Especially the association to eGFR highlights the importance for future studies to address renal function as possible influence on betatrophin regulation and consider eGFR as potential confounder when analyzing the role of betatrophin in humans.

## Introduction

A recent report about a newly identified predominantly liver derived circulating factor referred to as betatrophin attracted great attention because of its capability to promote beta cell proliferation and improving glucose tolerance in mice [1]. Betatrophin also called ANGPTL8, RIFL or Lipasin was initially identified in 2012. It is an atypical member of the angiopoietin-like protein family (ANGPTLs) functionally considered as a nutritionally-dependent blood lipid regulator [2–4] inhibiting the lipoprotein lipase and thereby regulating triglyceride (TG) levels [5]. Subsequent studies analyzing the phenotype of betatrophin knock-out animals challenged the perspective of betatrophin having a prominent role in glucose metabolism. While knock-out animals showed clear signs of an altered lipid metabolism with significantly lower TG levels in the fed state no changes in glucose homeostasis were observed [6]. Nevertheless the identification of this liver derived circulating factor potentially involved in the regulation of glucose and lipid metabolism triggered a great research interest in this hormone as potential therapeutic target or new biomarker for the metabolic syndrome, leading to several clinical studies examining the role of betatrophin in humans with multiple pathological conditions. Most studies so far compared betatrophin levels between diabetic and non-diabetic subjects (type 2 diabetes, type 1 diabetes and gestational diabetes), providing quite heterogeneous results. While the majority showed higher serum concentrations for diabetic patients [7–9] others failed to detect any significant differences [10]or even showed decreased betatrophin levels for diabetic compared to non-diabetic individuals[11]. Based on the pre-clinical data indicating that betatrophin is a nutrition dependent lipogenic factor, appearing as a potential mediator of post-prandial fatty acid trafficking and storage to adipose tissue [4], a number of clinical studies investigated the role of betatrophin in lipid metabolism showing positive correlations with triglycerides [9, 12, 13]. Beyond that, the results of studies looking for a putative link to obesity presented a similar heterogeneity as in diabetes, differing for betatrophin levels to be higher [14], lower [15] or equal [16] comparing patients with obesity to lean individuals. Taking into consideration the partial contradictory results on the influence of betatrophin on glucose and lipid metabolism as well as obesity, we analyzed the participants of the Metabolic-Syndrome Berlin-Potsdam follow-up cohort with respect to their betatrophin levels in relation to markers of the metabolic syndrome. We included intima media thickness as a marker for atherosclerosis and CKD-EPI estimated GFR as a marker for renal function in order to analyze two further age dependent phenotypes, given the situation that the majority of clinical studies has pointed towards a strong positive correlation of betatrophin with age [8, 13, 17–19].

## Material and methods

For this study blood samples of 535 individuals of the Metabolic Syndrome Berlin Potsdam (MesyBePo) follow-up study were analyzed. Details of baseline phenotyping were described previously [20]. The study protocol was approved by the ethics committees of the Landesärztekammer Brandenburg and the Freie Universität Berlin. All study samples were taken at 8:00 a.m. after an overnight fast. An oral glucose tolerance test (OGTT) was performed in all individuals, and fasting blood samples were taken before OGTT. Patient classification with respect to impaired glucose tolerance (IGT), impaired fasting glucose (IFG) and T2DM was made according to either prior diagnosis of T2DM with accompanying antidiabetic medication or based on the results of fasting glucose and 2h glucose levels after GTT according to the official WHO diagnostic criteria [21]. EDTA and citrate plasma as well as serum was taken from participants. Plasma was placed on ice and spun down within 30 min at 4°C. After centrifugation, the plasma was immediately transferred to a −20°C freezer. Within the next 24 h, all samples were transferred on dry ice to a −80°C freezer, where aliquots were stored until assay. Serum was left at room temperature for 30 min to allow clotting, thereafter, the sample was centrifuged. The serum was then transferred to a −20°C freezer and within 24 h to a −80°C freezer, comparable to all plasma samples. Betatrophin levels were measured by validated enzyme-linked immunosorbent assay (ELISA) kit provided by EIAAB (Catalogue number E1164H; Wuhan, China). The internal quality control revealed an intra-assay coefficient of variability of 5.2% and an inter-assay CoV of 13.6% for the measured full-length betatrophin levels in plasma samples. Carotid arterial IMT was measured at the posterior wall of the common carotid artery at three different positions using a high resolution ultrasound (Kretz AG, Germany). Mean and maximum values for IMT, which included possible plaques, were calculated. CKD-EPI estimated glomerular filtration rate was calculated according to the established mathematical formula from the Chronic Kidney Disease Epidemiology Collaboration [22].The study was carried out in accordance with the recommendations of the Declaration of Helsinki, and the experimental protocol of the study was approved by the institutional ethical committee. All subjects gave written informed consent. Statistical calculations were performed using SPSS 22.0 software (IBM Statistics, Germany). All values are presented as mean and SEM, if not otherwise mentioned. Logarithmic transformation of betatrophin levels was performed to achieve normal distribution tested by visual inspection and Shapiro Wilk test. The statistical tests in general were performed with ln(betatrophin) values. To provide a better comparability and understanding non-transformed values were depicted when absolute plasma concentration were presented. One-way ANOVA was performed followed by post-hoc Bonferroni test. A value of p < 0.05 was considered as statistically significant. Pearson Correlation coefficients were analyzed with respect to log Betatrophin. Multivariate linear regression models were calculated to identify independent relations between Betatrophin and ageing, renal function and multiple markers of the metabolic syndrome. Three models were calculated: crude without adjustment, model 1 was adjusted for age and sex only and model 2 was additionally adjusted for waist circumference, systolic blood pressure, HbA1c, total cholesterol/HDL cholesterol ratio, triglycerides and smoking (fully adjusted model).

## Results

Betatrophin levels were analyzed in participants of the MesyBePo study follow-up cohort (Metabolic Syndrome Berlin-Potsdam) that included healthy control subjects as well as patients with impaired glucose metabolism including overt type 2 diabetes. Baseline Characteristics of the study cohort are summarized in Table 1.

**Table 1:** Baseline characteristics of the MesyBePo Cohort (N = 535) with respect to the included markers of the metabolic syndrome.

Baseline Characteristics		
	N = 535	S.E.M.
Male	151 (28.2%)	
Female	384 (71.8%)	
Age (years)	55.6	± 0.49
Hypertension	179 (33.5%)	
Blood Pressure systolic (mmHg)	126.5	± 0.74
Blood Pressure diastolic (mmHg)	78.4	± 0.42
Smoking	45 (8.4%)	
Coronary Heart Disease	19 (3.6%)	
Intima Media Thickness (mm)	0.73	± 0.01
Overweight	418 (78.1%)	
Body Mass Index	29.6	± 0.26
Waist Circumference (cm)	96.4	± 0.59
Dyslipoproteinemia	113 (21.1%)	
Cholesterol (mmol/l)	5.53	± 0.04
HDL (mmol/l)	1.43	± 0.02
LDL (mmol/l)	3.45	± 0.04
Triglycerides (mmol/l)	1.45	± 0.03
Type 2 Diabetes	32 (6.0%)	
HbA1c (%)	5.5	± 0.03
HbA1c (mmol/mol)	37.1	± 0.30
Fasting Glucose (mg/dl)	98	± 0.95
Glucose 2h after oGTT (mg/dl)	120	± 2.02
ASAT (IU/l)	26.1	± 0.46
ALAT (IU/L)	19.4	± 0.46
GGT (IU/L)	26.0	± 0.68
Creatinine (mg/dl)	0.89	± 0.01
CKD-EPI eGFR (ml/min)	81.2	± 0.68
Betatrophin (pg/ml)	543.1	± 10.5

In a crude analysis (Table 2) with respect to different markers of the metabolic syndrome, we found a strong positive correlation of serum betatrophin concentrations with age (r = 0.47; p <0.001) as it has been described previously ^8, 13, 17–19^. Furthermore serum levels of betatrophin were positively correlated to markers of glucose metabolism HbA1_c_ (r = 0.24; p < 0.001) as well as fasting glucose levels (r = 0.20; p < 0.001) and serum glucose concentration 2h after performing a standard oral glucose tolerance test (r = 0.15; p < 0.001). In accordance calculated indices for insulin sensitivity HOMA-IR (r = 0.19; p < 0.001) and QUICKI (r = -0.14, p = 0.001) showed similar associations. Comparing the different subgroups of our cohort with respect to an altered glucose metabolism, we see the same constellation as it has been previously published [7, 13, 18]. We found participants with diabetes having significantly higher betatrophin levels than individuals having a normal glucose metabolism, with no significant differences for people with an impaired fasting glucose (IFG), impaired glucose tolerance (IGT) or both (IFG+IGT) compared to controls (One-way ANOVA p < 0.001; mean betatrophin concentration: controls = 506.4 pg/ml, IFG = 601.1 pg/ml (p = 0.42), IGT = 554.6 pg/ml (p = 1.0), IFG+IGT = 643.2 pg/ml (p = 0.873), type 2 diabetes 659.9 pg/ml (p < 0.001)). In our study we found no correlations with parameters of cholesterol metabolism, total cholesterol (r = -0.01; p = 0.83), LDL cholesterol (r = -0.06; p = 0.191) or HDL cholesterol (r = -0.02; p = 0.636) but a positive correlation with triglyceride levels (r = 0.12; p = 0.007). With respect to obesity we found a positive correlation of betatrophin with waist circumference (r = 0.23; p < 0.010) and with participants body mass index (r = 0.12; p = 0.008). Interestingly we found a distinct negative correlation of betatrophin with CKD-EPI eGFR (r = -0.33; p < 0.001). The majority of participants from the MeSyBePo cohort showed a normal to moderately decreased renal function (GFR category 1 | > 90ml/min: 27.8%, GFR category 2 | 60–89 ml/min: 63.1%, GFR category 3 | 30–59 ml/min: 6.0%). In correspondence to the mentioned association the comparison of betatrophin levels with respect to the usual GFR categories revealed significant differences between each standard GFR category with the highest betatrophin level in the bottom GFR group: Betatrophin—GFR category 1: 469.3 pg/ml (S.E.M. 16.3), GFR category 2: 550.4 pg/ml (S.E.M. 11.3), GFR category 3: 799.0 pg/ml (S.E.M. 79.5), p < 0.001. Since no patients with an eGFR < 30 ml/min participated in our study, we are unfortunately not able to address the role of betatrophin in patients with a severely impaired renal function. Additionally intima media thickness as marker for arteriosclerosis showed a positive correlation with betatrophin (r = 0.26; p < 0.001) as well systolic blood pressure (r = 0.17; p < 0.001). The positive correlation of betatrophin (β = 0.261, p < 0.001) with the intima media thickness was not found when adjusting for age, sex and cardiovascular risk factors.

**Table 2:** Correlation coefficients and corresponding p-values.

Correlations	ln Betatrophin	
Variable	R	p-value
Age	**0.469**	**< 0.001**
Intima Media Thickness	**0.261**	**< 0.001**
CKD-EPI eGFR	**-0.329**	**< 0.001**
Creatinine	**0.277**	**< 0.001**
Systolic Blood Pressure	**0.171**	**< 0.001**
Diastolic Blood Pressure	0.014	0.758
BMI	**0.115**	**0.008**
Waist Circumference	**0.227**	**< 0.001**
Total Cholesterol	-0.009	0.830
LDL Cholesterol	-0.057	0.191
HDL Cholesterol	-0.021	0.636
Triglycerides	**0.116**	**0.007**
HbA1c	**0.235**	**< 0.001**
Fasting Glucose	**0.200**	**< 0.001**
Glucose 2h after OGTT	**0.150**	**0.001**
ASAT	0.064	0.141
ALAT	0.055	0.205
Smoking	0.073	0.093

Notably the negative correlation for renal function remained associated with betatrophin (β = -0.197, p< 0.001) after adjustment for age, gender, abdominal obesity and standard parameters of lipid and glucose metabolism in a multivariate linear regression model (Table 3).

**Table 3:** Linear regression for Betatrophin in the fully adjusted model with representation of significant standardized beta value.

ln Betatrophin				
Parameter	Correlation	Standardized Beta	Correlation x Standardized Beta x 100	p-Value
**Age**	**0.301**	**0.322**	**9.70**	**< 0.001**
**CKD-EPI eGFR**	**-0.194**	**-0.197**	**3.81**	**< 0.001**
**Gender**	**-0.157**	**-0.150**	**2.34**	**< 0.001**
**Waist Circumference**	**0.123**	**0.121**	**1.49**	**0.006**
**Total/HDL Cholesterol Ratio**	**-0.095**	**-0.096**	**0.91**	**0.033**
HbA1c	0.071	0.066	0.47	0.112
Triglycerides	0.055	0.056	0.31	0.217
Systolic Blood Pressure	-0.028	-0.027	0.07	0.533
Smoking	0.001	0.001	0.00	0.986
**Total**			**19.11**	**< 0.001**

The multiplicative term (Correlation x Standardized β x100) explains the variation of betatrophin explained by the respective parameter in percent.

## Conclusion

The initial hope to use betatrophin as drug therapy target for diabetec patients following the discovery that it was potentially able to promote beta-cell proliferation in mice[1] has not been fulfilled so far. On one hand subsequent preclinical studies examining the effects of deletion as well as overexpression of betatrophin showed no evidence for a prominent isolated role of betatrophin in controlling beta-cell proliferation[23, 24]. On the other hand the clinical evaluation of betatrophin in humans yielded a quite heterogeneous pattern with partially contradictory results [11, 18, 25, 26]. The recent retraction of the initial paper describing betatrophin as potential beta cell mitogen [27] and the collected data from clinical studies largely invalidate the perspective of a clinically relevant player in beta cell proliferation. Nevertheless the so far accumulating data on betatrophin suggest a more intermingled role in metabolic function potentially involving the cross talk of lipid and glucose regulation [28]. In our analysis we found betatrophin to be positively correlated to HbA1c, fasting blood glucose and triglyceride levels as well as BMI and waist circumference. This is in line with the majority of the so far published clinical studies. One important aspect addressing the divergent results for betatrophin in humans might be the use of different ELISA kits for detecting circulating betatrophin levels. In this analysis we used the commercially available ELISA kit provided by EIAAB (Catalogue number E1164H; Wuhan, China) which is based on an antibody recognizing the N-terminus and is the most commonly used detection method so far [8, 13, 17, 19, 25, 29, 30]. A recent report compared this ELISA kit to a second commonly used kit from Phoenix Pharmaceuticals (Catalogue number EK-051-55; Burlingame, CA, USA) that is based on an antibody against the C-terminus [31]. While both assays adequately recognized human recombinant betatrophin the results indicate that betatrophin undergoes proteolytic regulation releasing the C-terminal fragments. As consequence the different ELISAs measure either full-length betatrophin only or full-length protein and C-terminal fragment. It is currently not known if either full-length betatrophin or the C-terminal fragment is responsible for the biological effect nor what further processes are involved in betatrophin degradation and clearance. Nevertheless this discrepancy may in part explain the largely diverse results of the clinical studies conducted so far. Numerous different ELISA kits with variable technical properties for detecting the different protein subsets have been used for the analysis of the published clinical data. This aspect points out particularly that great caution is advised for the interpretation and comparison of betatrophin levels and the proteins potential relevance in metabolic regulation. We recently published a study on a potential role of betatrophin in the postprandial switch from lipid to glucose metabolism showing that the impaired betatrophin response in obese subjects is restored after weight loss to a degree comparable to lean individuals [28]. For this trial we used the same ELISA kit for detecting betatrophin further prompting the notion of the full length protein might be the active form of betatrophin. Given betatrophin’s putative dual role in lipid and glucose metabolism as well as its correlation with ageing and in some studies towards an atherogenic cholesterol profile [25, 30], it is tempting to investigate a possible influence on markers of arteriosclerosis. In our study we see no correlation of betatrophin with total, LDL or HDL cholesterol but a positive correlation with systolic blood pressure and intima media thickness. These associations were only detectable in the crude analysis and disappeared when adjusting for age and gender making an independent betatrophin effect less likely. Interestingly, even after fully adjusting for cardiovascular risk factors age, gender, waist circumference, total/HDL cholesterol ratio and renal function remain significantly correlated with betatrophin. Similar to the above mentioned results correlations of betatrophin and age can be found in recent clinical studies to be either positive [18, 19] or negative [32]. With respect to sex specific differences betatrophin levels have been described to be significantly higher in males [16] as well as in females [11]. Besides differences in detection methods other factors might be responsible for the discrepancies. The results come from different study populations differing largely in age, ethnicity, basic disease state and medication. In our cohort adjusting for the intake of antihypertensive, lipid lowering or antidiabetic medication has no substantial impact on the described associations while only antidiabetic drug treatment exerts in independent influence in the fully adjusted model (std. ß = 0.143, p = 0.001). Given the low prevalence of antidiabetic drug treatment (2.8% of all participants) it remains elusive if a distinct pharmacological influence on betatrophin need to be taken into account in general. Notably, data on a possible influence of renal function on circulating betatrophin levels are sparse, taking into consideration that study populations were varying largely by age and are affected by different manifestations of the metabolic syndrome that predispose for an impaired renal function. Some smaller studies identified weak positive correlations with serum creatinine levels not reaching statistical significance [8, 13, 32]. One recent Japanese study in diabetic patients found a significant negative correlation of betatrophin and creatinine clearance as well as eGFR (calculation adjusted for Japanese population) that seemed mainly driven by a common association of the two factors with age [19]. In slight contrast a recent German study, analyzing betatrophin levels in diabetic patients undergoing hemodialysis compared to diabetic individuals with sustained renal function, found a significant positive correlation of betatrophin and eGFR [33]. In this study patients on hemodialysis had significantly lower betatrophin levels compared with subjects having an eGFR > 50ml/min/1,73m^2^. A finding that may argue against a possible renal elimination of betatrophin, that has been the initial hypothesis of the authors. Furthermore a third recent study from Taiwan investigated the correlation of full-length betatrophin with urinary albumin excretion and eGFR as markers of diabetic nephropathy in patients suffering from type 2 diabetes. They found serum betatrophin levels to be higher in patients with macroalbuminuria compared to normoalbuminuria with an independent inverse association to eGFR and speculate betatrophin to be a novel endocrine regulator involved in the progression of diabetic nephropathy [34]. Unfortunately our data acquisition did not include albumin excretion in 24h urine nor urine creatinine levels. Solemnly albumin concentration in the spot urine was measured and didn’t reveal a significant correlation with betatrophin. Given the low prevalence of elevated urinary albumin excretion in our cohort (albumin concentration < 20 mg/l: 86.0%; albumin concentration 20–200 mg/l: 13.3%; albumin concentration > 200 mg/l: 0.7%), we cannot properly address the aspect of an association between betatrophin and urinary albumin excretion or albumin/creatinine ratio as additional markers for kidney function. Nevertheless in the study by Chen et. al. elevated betatrophin levels in type 2 diabetes have been found to be associated with impaired renal function and also with urinary albumin excretion. The same detection method for full-length betatrophin has been applied there and the findings have been interpreted as potential link of betatrophin function with respect to lipid metabolism and the progression of diabetic nephropathy. In our own cohort consisting of 535 patients from the MesyBePo follow-up study with a normal to mildly impaired renal function (average eGFR of 81.2 ml/min at an average of 55.6 years; 6% of participants diagnosed with diabetes) betatrophin showed a similarly strong independent correlation of circulating hormone levels and renal function. Interestingly for other members of the angiopoietin-like protein family such as ANGPTL3, on which betatrophin has a regulatory effect by promoting ANGPTL3 cleavage, a higher serum concentration in patients with kidney disease has been described [35]. The same phenomenon of elevated blood concentrations in patients with impaired renal function has been documented for a number of other hepatokines and adipokines e.g. FGF21 [36], chemerin [37] and leptin [38]. Taken together circulating betatrophin levels are independently associated with renal function. Our study further extends the data on betatrophin’s potential role in diabetic nephropathy in terms of linking betatrophin to renal function even in absence of a decisive renal pathology. At this point it remains an open question if betatrophin exerts an effect on kidney function or the progression of a pre-existing nephropathy or if wise versa betatrophin turnover depends on renal function. Further experiments are needed to elucidate physiological significance of this finding and future studies on betatrophin should take a possible influence of renal function into special account.
